# Comparison of profiles of biomarkers of potential harm among healthy adults who used heated tobacco products, smoked combustible cigarettes, or who had never smoked: a cross-sectional, observational study

**DOI:** 10.1007/s11739-025-04202-z

**Published:** 2025-11-26

**Authors:** Daisuke Nishihara, Dai Yuki, Naoki Minami

**Affiliations:** Scientific and Regulatory Affairs, Japan Tobacco Inc., 4-1-1 Toranomon, Minato-Ku, Tokyo 105-6927 Japan

**Keywords:** Heated tobacco products, Combustible cigarettes, Harmful and potentially harmful constituents, Biomarkers of potential harm, Observational study

## Abstract

This cross-sectional observational study assessed exposure to a selected combustible cigarette (CC) smoke constituent and biomarkers of potential harm (BoPH) in healthy adults who exclusively used a heated tobacco product (direct heating tobacco system, platform 3, generation 3, version a [DT3.0a] group, *n* = 304), smoked CC (CC group, *n* = 102), or had never smoked CC (NS group, *n* = 102), to evaluate biomarker profiles relevant to health risks associated with smoking. Relative to the CC group, exposure to 4-(methylnitrosamino)-1-(3-pyridyl)-1-butanone was 39.1% lower in the DT3.0a group (*p* < 0.01), and several BoPH were lower in the DT3.0a group, including 2,3-dinor-thromboxane-B2 (− 19.4%, *p* = 0.04), 11-dehydrothromboxane-B2 (− 10.2%, *p* = 0.25), 8-epi-prostaglandin-F2α (− 7.2%, *p* = 0.42), and white blood cell count (− 11.6%, *p* = 0.05). Relative to the CC group, high-density lipoprotein cholesterol was 32.0% higher in the DT3.0a group (*p* < 0.01) with pulmonary function parameters also being higher, although the differences were not statistically significant. For these biomarkers, the NS group exhibited trends similar to those observed in the DT3.0a group. In contrast to previous findings, soluble intercellular adhesion molecule-1 levels were similar between the CC and NS groups and highest in the DT3.0a group, suggesting that further assessments may be more appropriate in the context of change in values. Except for this point, the DT3.0a group showed BoPH profiles trending in the direction toward those of the NS group, suggesting that the health risks associated with smoking are potentially lower in DT3.0a use compared to CC smoking.

**Trial Registration : **Prior to the recruitment, the study was registered at the UMIN Clinical Trials Registry on December 27, 2022(UMIN000049840).

**Supplementary Information:**

The online version contains supplementary material available at 10.1007/s11739-025-04202-z.

## Introduction

Cigarette smoking is a cause of diseases including lung cancer, coronary heart disease, emphysema and chronic bronchitis [1]. It is reported that the development of diseases associated with smoking is primarily due to the repeated and long-term exposure to harmful constituents generated from burning tobacco while smoking combustible cigarettes (CCs) [1, 2]. Recently, the market for non-combustible tobacco products, such as heated tobacco products (HTPs), has been growing. These have the potential to reduce the health risks associated with smoking by reducing the exposure to the harmful and potentially harmful constituents (HPHCs) associated with burning tobacco. For HTP, a number of clinical studies have reported that switching from CC to HTP leads to substantial and sustained reductions in exposure to the selected HPHCs [2]. Available clinical evidence indicates that reducing exposure to the HPHCs in cigarette smoke has the potential to reduce the health risks associated with smoking [3–5], although further evidence is required to characterize biologically meaningful changes.

Although there is no clear consensus on how to generate scientific evidence demonstrating measurable and substantiated reductions in health risks from HTP use, the U.S. Food and Drug Administration requires scientific evidence that shows a significant reduction in disease-related morbidity and mortality associated with tobacco. This typically involves conducting research including long-term epidemiological studies with HTP to support a risk modification claim [6, 7]. As the development of chronic diseases associated with smoking typically involves long latency periods (e.g., decades), measuring such outcomes in clinical studies that are typically shorter in duration is challenging. From this perspective, assessing surrogate biomarkers of disease associated with smoking, so-called biomarkers of potential harm (BoPH), has been adopted as a scientific approach to evaluate likely long-term health outcomes associated with HTP use [8–10]. Suitable biological indicators to serve as BoPH have been extensively reviewed and proposed (e.g., relationship with smoking and disease associated with smoking, reversibility following smoking cessation, etc.) [8–10], and are widely used to characterize tobacco and nicotine-containing products in relation to cardiovascular and/or pulmonary disease risks. These include indices such as inflammation, oxidative stress, arterial endothelial function, lipid metabolism, and physiological pulmonary functions [11–14]. Although long-term epidemiological studies may be more suitable for providing an answer as to whether HTP use is associated with a reduction in health risks, the current BoPH assessments may at least indicate that there is no increased health risk in the short term [3, 12]. Furthermore, meaningful changes in relevant BoPH have been reported in studies over a short duration of time that provide evidence of potential reduction in disease risks associated with smoking [9] benefits for providing parties in the present days such as tobacco product users, science communities and regulators with evidence for potential reduction in the health risks associated with smoking.

The direct tobacco heating system platform 3 generation 0 version a (DT3.0a) is an HTP developed by Japan Tobacco Inc. and has been commercially available in Japan since 2021 under the brand “Ploom X”. As a result of heating and not burning tobacco, chemical analyses have shown that the levels of the selected HPHCs [15] in DT3.0a aerosol are significantly lower than in the smoke of the University of Kentucky reference cigarette (1R6F) [16]. In addition, a 5-day confinement study in healthy adults who smoked showed that switching from CC to DT3.0a resulted in substantial reductions in exposure to the selected HPHCs compared with continued CC smoking, with reductions comparable in magnitude to those observed in the smoking abstinence group [17]. These characteristics led researchers to conclude that the DT3.0a product, when used by adults who smoke, has the potential to reduce the health risks associated with smoking.

Building on these previous studies, the current study aimed to gain further insights into the potentially lower health risks associated with smoking in the DT3.0a use under real-world actual-use conditions, by assessing relevant BoPH levels and comparing them with those in smoking CC and never-smoking CC. An observational, cross-sectional study was conducted in healthy adults in which the target population was DT3.0a users who have used the product exclusively for more than 3 months. For the control groups, adults who had exclusively smoked CC for more than 1 year and adults who had never smoked were selected. To assess exposure levels, a biomarker of exposure (BoE) to a tobacco-specific nitrosoamine present in CC smoke was also measured. For adults who smoke, a dose–response relationship has been reported between BoPH levels and daily CC consumption, especially for BoPH found in blood and urine [6]. Since DT3.0a generates an aerosol with fewer and lower levels of HPHCs than CC, the amount of daily DT3.0a consumption may be less relevant in relation to BoPH levels. For this reason, an exploratory analysis was performed to characterize the dose–response relationship between BoPH levels and daily product consumption for the CC and DT3.0a groups using linear regression analysis.

## Methods

### Study design

This cross-sectional observational study was conducted in three groups: DT3.0a, CC, and NS groups consisting, respectively, of adults who daily and exclusively used DT3.0a, who daily and exclusively smoked CC, and who had never smoked CC at any time in their lives. The study was conducted at three study sites in Japan: SOUSEIKAI Sumida Hospital, Tokyo; SOUSEIKAI Nishikumamoto Hospital, Kumamoto; and SOUSEIKAI PS Clinic, Fukuoka.

Age, sex, and body mass index (BMI) were considered to be factors that could potentially impact BoPH levels [18, 19]. Imbalance between the groups in terms of the demographic composition of these factors may jeopardize appropriate intergroup comparison, therefore, the present study involved matching of participants. First, we enrolled participants into the DT3.0a group and calculated the composition ratio of the 12 sub-groups characterized by a combination of age (30–39, 40–49 or 50–64 years), sex (male or female), and BMI (< 25.0 or ≥ 25.0) in the DT3.0a group. Then, recruitment was performed for the CC and NS groups such that the 12 sub-groups have the same composition ratio (with margin of ± 5%) as the DT3.0a group.

The screening and all study-related procedures were performed on the same day as a single ambulatory visit (screening and enrollment visit). Participants were informed of the study details, and those who agreed to participate in the study provided signed informed consent and submitted the first-void urine sample collected at home on the morning of the screening and enrollment visit (a laboratory kit for urine collection had been delivered in advance). Consented participants were checked for eligibility such as history of tobacco use, exhaled carbon monoxide (CO) concentration (only for the DT3.0a and NS groups), and urinary cotinine. Urinary human chorionic gonadotrophin testing was conducted in females to check for the possibility of pregnancy. Eligible participants were enrolled into the study and underwent the specified tests and examinations (sampling for biomarkers, respiratory function test, questionnaire etc.). Blood sampling and spirometry measurement were performed in the morning or afternoon depending on visit schedules.

### Participants (inclusion and exclusion criteria)

Participants in the present study were volunteers, of Japanese ethnicity, male or female aged 30–64 years, residing in Japan and recruited from a clinical trial volunteer panel. Main inclusion criteria were as follows: DT3.0a group, participants who had used DT3.0a exclusively on a daily basis (more than 4 days per week) for 3 months or more in advance to the screening and enrollment visit and whose level of exhaled CO was ≤ 10 ppm (measured by a piCO™ Advance Smokerlyzer® [Bedfont Scientific Ltd, Maidstone, England]) and who tested positive for urinary cotinine with 200 ng/mL as the cut-off value (a similar method was applied in the previous study [4]); CC group, participants who had smoked CC exclusively on a daily basis (more than 4 days per week) for 1 year or more in advance to the screening and enrollment visit and who self-reported to have smoked more than 100 cigarettes over their lifetime and who tested positive for urinary cotinine; NS group, participants who had never used any kind of tobacco or nicotine-containing products (using even one unit of such product was grounds for exclusion) and whose level of exhaled CO was ≤ 10 ppm and who tested negative for urinary cotinine (cut-off 200 ng/mL). Main exclusion criteria were female participants who were pregnant or breastfeeding, participants who tested positive for hepatitis virus B, hepatitis virus C, and/or human immunodeficiency virus, and/or syphilis infection (serological test), participants who had donated ≥ 200 mL of blood within 4 weeks or donated ≥ 400 mL of blood within 12 weeks (males) or 16 weeks (females) in advance to the screening and enrollment visit, and participants who had any clinically relevant abnormal findings (current diagnosis or history of malignancy, serious cardiovascular, liver, renal, hematologic and respiratory diseases, and serious endocrine, metabolic and electrolyte abnormality/disorder, as well as having positive result for the SARS-CoV-2 antigen test) as judged by the investigators. Participants were also excluded if they had used any prescription drugs, over-the-counter medications (including smoking-cessation medications), or dietary supplements within 2 weeks in advance to the screening and enrollment visit.

### Demographics and other characteristics

The baseline characteristics of all participants including age, sex, and BMI were recorded. History of tobacco product use, daily CC or HTP consumption, and frequency of product use were recorded for participants in the DT3.0a and CC groups. The NS group participants were also asked about secondhand exposure to either aerosol or smoke from tobacco products. In addition, nicotine equivalents (calculated as the molar sum of nicotine and its five major metabolites) were measured in urine to assess the nicotine uptake levels, using validated LC–MS/MS methods at the Celerion Laboratories (Lincoln, NE, USA) [20]. The nicotine equivalent values were adjusted by the urinary creatinine levels.

### Study products

No study product was provided by the sponsor or study investigator.

### Biomarkers

As an assessment for BoE, 4-(methylnitrosamino)−1-(3-pyridyl)−1-butanol and its glucuronides, NNAL-O-glucuronide and NNAL-N-glucuronide (total NNAL) in urine were analyzed to assess exposure to 4-(methylnitrosamino)−1-(3-pyridyl)−1-butanone (NNK). Total NNAL and creatinine in first-void urine were measured by LC–MS/MS using validated methods at the Celerion Laboratories (Lincoln, NE, USA) [20]. The total NNAL values were adjusted by the urinary creatinine levels.

The selected BoPH assessed in this study have been shown to indicate early changes in biological and physiological functions underlying clinical symptoms and disease development relevant to diseases associated with smoking [8–10]. A list of the BoPH assessed in the present study is presented in Table 1. Urine BoPH included 11-dehydrothromboxane B2 (11-DHTXB2) and 2,3-dinor thromboxane B2 (2,3-d-TXB2) as biomarkers for platelet activation and 8-epi-prostaglandin F2α (8-epi-PGF2α) as a biomarker for oxidative stress. These urine BoPH were reported to be lower in never-smokers compared to CC smokers, and to decrease following smoking abstinence [8–10]. Blood BoPH included high-density lipoprotein cholesterol (HDL-C) as a biomarker for lipid metabolism, where higher HDL-C levels have been observed in never-smokers compared to CC smokers as well as following smoking cessation [8–10]. Blood BoPH also included soluble intercellular adhesion molecule-1 (sICAM-1) and white blood cell count (WBC), both of which are related to inflammation. Lower levels of sICAM-1 and WBC have been reported for never-smokers compared to CC smokers, with smoking abstinence leading to decreases in these BoPH [8–10]. 11-DHTXB2, 2,3-d-TXB2 and 8-epi-PGF2α were measured in first-void urine by LC–MS/MS with validated methods at Celerion Laboratories (Lincoln, NE, USA), and the values of 11-DHTXB2, 2,3-d-TXB2 and 8-epi-PGF2α were adjusted by the urinary creatinine levels. HDL-C was measured in serum by colorimetric assays. sICAM-1 was measured in plasma by immunoassay (Human sICAM-1 ELISA Kit [Thermo Fisher Scientific Inc., Waltham, MA, USA]). WBC was determined in whole blood samples by flow cytometry. Measurements of HDL-C, sICAM-1, and WBC were conducted with validated methods at LSI Medience Inc. (Tokyo, Japan).

**Table 1:** List of biomarkers of potential harm

Matrix/physical test	Biomarkers of potential harm	Acronym	Relevant biological mechanisms	Relevant smoking-related diseases
Blood	High-density lipoprotein cholesterol	HDL-C	Lipid metabolism	Cardiovascular diseases
	Soluble intercellular adhesion molecule-1	sICAM-1	Vascular inflammation	Cardiovascular diseases
	White blood cell count	WBC	Inflammation	Cardiovascular diseases
Urine	11-Dehydrothromboxane B2	11-DHTXB2	Platelet activation	Cardiovascular diseases
	2,3-Dinor Thromboxane B2	2,3-d-TXB2	Platelet activation	Cardiovascular diseases
	8-Epi-Prostaglandin F2α	8-epi-PGF2α	Oxidative stress	Cardiovascular diseases
Pulmonary function	Percent predicted forced vital capacity	%FVC	Pulmonary functions	Pulmonary diseases
	Forced expiratory volume in one second	FEV1	Pulmonary functions	Pulmonary diseases
	Percent predicted forced expiratory volume in one second	%FEV1	Pulmonary functions	Pulmonary diseases
	Percent forced expiratory volume in one second	FEV1%	Pulmonary functions	Pulmonary diseases
	Forced expiratory flow between 25 and 75% of forced vital capacity	FEF_25–75_	Pulmonary functions	Pulmonary diseases
	Peak expiratory flow	PEF	Pulmonary functions	Pulmonary diseases

As the BoPH for pulmonary function, parameters including forced expiratory volume in one second (FEV1), percent predicted value of FEV1 (%FEV1), forced vital capacity (FVC), percent predicted value of FVC (%FVC), percent forced expiratory volume in one second (FEV1%), forced expiratory flow at 25–75% of FVC (FEF_25–75_), and peak expiratory flow (PEF) were measured without a bronchodilator at the clinics using a spirometer based on the ATS/ERS guidelines [21]. Among pulmonary function variables, %FEV1 is mainly used for the diagnosis of chronic obstructive pulmonary disease (COPD), while a few epidemiological studies have shown higher FEV1 and FEF_25–75_ in never-smokers compared to CC smokers [8–10, 22].

### Questionnaire

To characterize the status of cough-related symptoms, a validated Japanese version of the Leicester cough questionnaire (LCQ) [23], J-LCQ, was administered to participants [24]. The J-LCQ consists of 19 items rated on a 7-point Likert scale. A higher score indicates a better health status in relation to cough. The 19 items are grouped into three domains: physical, psychological, and social, with each item being scored and the scores are then summed to provide domain scores and a total score. In the present study, the scores for each domain and their total score (LCQ) were calculated. Permission to use the J-LCQ in the present study was obtained from its developer, Dr. Birring and translators, Drs. Niimi and Ogawa.

### Statistical analysis

As no data were available on BoPH levels during daily use of the DT3.0a over a defined period under real-world conditions, sample size considerations were guided by findings from earlier studies [8, 9, 11]. For commonly reported BoPH, including WBC and 8-epi-PGF2α, assuming a two-sided *t* test and a 5% type I error rate, a sample size of 36–82 participants per group was estimated to provide at least 80% power to detect a difference. For 11-DHTXB2, under the same assumptions, a sample size of 76–213 participants per group was required to achieve comparable statistical power. Given the variability across studies and individual BoPH, it was considered challenging to ascertain an appropriate sample size for evaluating statistical differences in BoPH. Therefore, the present study focused on comparing the overall profiles of various BoPH among the study groups, rather than testing statistical hypotheses for individual BoPH. Accordingly, no formal sample size calculation was performed. The target sample size was set at 300 participants for the DT3.0a group and 100 participants each for the CC and NS groups, in line with the original plan for a similarly designed cross-sectional observational study involving HTP users [9].

For BoE and BoPH, an analysis of covariance (ANCOVA) was performed for statistical comparison between the study groups. Log-transformed biomarker values were applied to achieve a normal distribution. The ANCOVA model included group, age, sex, BMI, and site as well as interaction of group and age, group and sex, group and BMI, and group and site. In the ANCOVA, sampling timing (morning or afternoon) and interaction between group and sampling timing were applied only for the models of blood BoPH, since the potential impact of diurnal variation was a concern.

Linear regression analysis was conducted for determining the relationship between BoPH values and consumption (number of sticks used or CC smoked per day for DT3.0a and CC, respectively) as an exploratory exercise.

Descriptive statistics were presented to describe the demographic and other characteristics (tobacco product use, nicotine uptake and inhalation of an aerosol or smoke from tobacco products used by the others), biomarkers, and cough assessment results. The two-sided significance level was set to 0.05. SAS for Windows (SAS Institute, Cary, NC) was used for conducting the statistical analyses.

For cases where the measured biomarker value was below the limit of quantification, a value half the limit of quantification was applied for the analysis. No imputation was performed for missing data. Complete case analysis was not performed.

## Results

### Participant disposition

Informed consent was obtained from 534 participants, with 508 participants enrolled into the study, comprising 304, 102, and 102 participants in the DT3.0a, CC, and NS groups, respectively (Fig. S1). The study was completed by 292 participants in the DT3.0a group, 95 in the CC group, and 92 in the NS group. The main reasons for withdrawal were positive serological test results, as well as bundle branch block or high blood pressure (the decision was made by the investigator for safety reasons in pulmonary function test).

### Demographics

A summary of participant demographics is shown in Table 2. The mean (standard deviation [SD]) age was 47.0 (8.2), 46.2 (8.0) and 46.5 (8.2) years, and the mean (SD) BMI was 24.2 (4.4), 24.4 (4.6), and 24.2 (3.9) kg/m^2^ for the DT3.0a, CC, and NS groups, respectively. The proportions of males were 88.5, 87.3, and 86.3% for the DT3.0a, CC, and NS groups, respectively. These demographic characteristics did not differ significantly among the study groups.

**Table 2:** Demographic summary

		**DT3.0a group**	**CC group**	**NS group**	**Total**
	*N*	304	102	102	508
Age (years)	Mean (SD)	47.0 (8.2)	46.2 (8.0)	46.5 (8.2)	46.7 (8.1)
	Median (min., max.)	48 (30, 64)	47 (31, 62)	48 (30, 64)	47 (30, 64)
Proportion in each group	30–39 years (*n*, %)	67 (22.0)	25 (24.5)	23 (22.5)	115 (22.6)
	40–49 years (*n*, %)	108 (35.5)	39 (38.2)	33 (32.4)	180 (35.4)
	50–64 years (*n*, %)	129 (42.4)	38 (37.3)	46 (45.1)	213 (41.9)
Gender (*n*, %)	Male	269 (88.5)	89 (87.3)	88 (86.3)	446 (87.8)
	Female	35 (11.5)	13 (12.7)	14 (13.7)	62 (12.2)
BMI (kg/m^2^, %)	Mean (SD)	24.2 (4.4)	24.4 (4.6)	24.2 (3.9)	24.3 (4.3)
	Median (min., max.)	23.6 (15.2, 49.4)	23.9 (16.0, 37.7)	23.3 (18.1, 35.7)	23.5 (15.2, 49.4)
Proportion in each group	< 25.0 (*n*, %)	197 (64.8)	68 (66.7)	67 (65.7)	332 (65.4)
	25.0≦ (*n*, %)	107 (35.2)	34 (33.3)	35 (34.3)	176 (34.6)

*BMI* body mass index, *CC* combustible cigarette, *DT3.0a* direct heating tobacco system platform 3 generation 3 version a, *NS* never smokers, *SD* standard deviation

### Other participant characteristics

Other participant characteristics relevant to tobacco products are summarized in Table 3.

**Table 3:** Other subject characteristics

		***N***		**Mean (SD)**	**Median (min.–max.)**
**CC group**	Current product use status	98	Cigarette smoking duration (months)	297.1 (107.5)	300 (14–540)
			Daily cigarette consumption (cigarettes per day)	13.9 (6.2)	15 (3–40)
		95	Nicotine equivalents (mg/g creatinine)	7.1 (4.3)	6.2 (0.2, 24.5)
**DT3.0a group**	Current product use status	301	DT3.0a use duration (months)	13.8 (4.9)	15 (3–20)
			Daily DT3.0a consumption (sticks per day)	15.5 (7.7)	15 (4–80)
		291	Nicotine equivalents (mg/g creatinine)	8.0 (4.5)	7.3 (0.1, 30.1)
	HTP use history in a lifetime	240	Duration (months)	47.2 (23.9)	48 (3–161)
			Daily consumption (sticks per day)	15.2 (7.8)	15 (3–80)
	Cigarette smoking history in a lifetime	292	Duration (months)	254.8 (114.2)	244 (5–540)
			Daily consumption (cigarettes per day)	16.2 (7.1)	15 (2–50)
	Proportion of the last products used before starting DT3.0a use*	301	HTPs (%)	64.5	
			Combustible cigarettes (%)	45.7	
			Other tobacco products (%)	3.6	
			Smoking cessation (%)	2.0	
			No experience of tobacco product use (%)	0.7	
	Proportion of daily cigarette smoking experience in a lifetime	299	Yes (%)	98.0	
			No (%)	2.0	
**NS group**	Proportion of inhalation of a smoke or a vapor from tobacco products used by the others	92	Nicotine equivalents (mg/g creatinine)	0.2 (0.1)	0.1 (0.1, 0.5)
			Yes (%)	35.8	
			No (%)	64.2	

^*^Multiple answers allowed

*CC* combustible cigarette, *DT3.0a* direct heating tobacco system platform 3 generation 3 version a, *HTP* heated tobacco product, *NS* never smokers, *SD* standard deviation

Participants in the CC group self-reported current and exclusive CC use for an average of 297.1 months, with a mean consumption of 13.9 sticks per day, and their mean nicotine equivalent was 7.1 mg/g creatinine.

Participants in the DT3.0a group self-reported current and exclusive DT3.0a use for an average of 13.8 months, with a mean consumption of 15.5 sticks per day, and their mean nicotine equivalent was 8.0 mg/g creatinine. Immediately before starting exclusive use of DT3.0a, 64.5 and 45.7% of the DT3.0a group had used/smoked HTP and CC, respectively (multiple answers were permitted to allow for any self-report of poly-tobacco product use), while 0.7% had not formerly used any tobacco product. In terms of lifetime use, 98.0% of the DT3.0a group had smoked CC daily at some point prior to exclusively using DT3.0a. For the DT3.0a group, the lifetime tobacco use history showed an average HTP use duration of 47.2 months and a mean consumption (including DT3.0a) of 15.2 sticks per day, and an average CC smoking duration of 254.8 months with a mean consumption of 16.2 sticks per day.

In the NS group, 35.8% of participants reported inhaling smoke or aerosol from tobacco products used by the others in daily life as their current status. All participants in the NS group had nicotine equivalent values below the limit of quantification.

### Biomarkers of exposure

The results of participants’ exposure to NNK (total NNAL) are presented in Table 4, with geometric LS mean ratios from the statistical model used to compare relative exposure levels between the study groups.

**Table 4:** Statistical comparison of biomarkers of exposure and potential harm

**Matrix/physical test**	**Biomarker/parameter**	**Group**	***N***	**Geometric LS mean** (**95% CI**)		**Geometric LS mean ratio (%)** (**95% CI**)	***p* Value**		**Geometric LS mean ratio (%)** (**95% CI**)	***p* Value**
Urine	Total NNAL (ng/g creatinine)	DT3.0a	292	37.0 (31.0, 44.2)						
		CC	95	60.9 (45.8, 80.8)	DT3.0a/CC	60.9 (43.6, 85.0)	0.0037			
		NS	92	2.4 (1.8, 3.2)	NS/CC	4.0 (2.7, 5.9)	< 0.0001	DT3.0a/NS	1525.7 (1095.9, 2124.2)	< 0.0001
Blood	HDL-C (mg/dL)	DT3.0a	292	66.1 (62.8, 69.5)						
		CC	95	50.0 (44.8, 56.0)	DT3.0a/CC	132.0 (116.8, 149.2)	< 0.0001			
		NS	92	62.3 (57.4, 67.5)	NS/CC	124.4 (108.4, 142.7)	0.0019	DT3.0a/NS	106.1 (96.5, 116.7)	0.2208
	sICAM-1 (ng/mL)	DT3.0a	292	508.5 (480.0, 538.7)						
		CC	95	402.6 (354.7, 457.0)	DT3.0a/CC	126.3 (109.9, 145.2)	0.0011			
		NS	92	377.1 (344.1, 413.1)	NS/CC	93.7 (80.1, 109.5)	0.4101	DT3.0a/NS	134.9 (121.1, 150.3)	< 0.0001
	WBC (10^3/uL)	DT3.0a	292	6.2 (5.9, 6.5)						
		CC	95	7.0 (6.3, 7.9)	DT3.0a/CC	88.4 (78.1, 100.1)	0.0512			
		NS	92	5.6 (5.1, 6.0)	NS/CC	79.4 (69.0, 91.2)	0.0012	DT3.0a/NS	111.4 (101.2, 122.6)	0.0284
Urine	11-DHTXB2 (ng/g creatinine)	DT3.0a	292	722.8 (655.6, 797.0)						
		CC	95	805.1 (688.5, 941.3)	DT3.0a/CC	89.8 (74.7, 108.0)	0.2514			
		NS	92	629.5 (539.6, 734.4)	NS/CC	78.2 (62.8, 97.4)	0.0282	DT3.0a/NS	114.8 (95.7, 137.8)	0.1374
	2,3-d-TXB2 (ng/g creatinine)	DT3.0a	292	408.2 (366.1, 455.2)						
		CC	95	506.3 (425.2, 602.7)	DT3.0a/CC	80.6 (65.7, 99.0)	0.0402			
		NS	92	343.3 (289.1, 407.6)	NS/CC	67.8 (53.1, 86.6)	0.0019	DT3.0a/NS	118.9 (97.0, 145.7)	0.0949
	8-epi-PGF2α (ng/g creatinine)	DT3.0a	292	170.5 (154.9, 187.8)						
		CC	95	183.8 (157.6, 214.5)	DT3.0a/CC	92.8 (77.3, 111.3)	0.4168			
		NS	92	140.7 (120.9, 163.8)	NS/CC	76.5 (61.6, 95.0)	0.0155	DT3.0a/NS	121.2 (101.3, 145.1)	0.0362
Pulmonary functions	%FVC (%)	DT3.0a	298	102.4 (99.7, 105.1)						
		CC	97	99.0 (94.9, 103.3)	DT3.0a/CC	103.4 (98.4, 108.6)	0.1875			
		NS	92	100.9 (96.8, 105.2)	NS/CC	101.9 (96.0, 108.1)	0.5325	DT3.0a/NS	101.5 (96.6, 106.6)	0.5624
	FEV1 (L)	DT3.0a	298	2.9 (2.8, 3.0)						
		CC	97	2.7 (2.6, 2.9)	DT3.0a/CC	106.2 (99.8, 113.0)	0.0573			
		NS	92	2.8 (2.7, 3.0)	NS/CC	103.7 (96.3, 111.7)	0.3375	DT3.0a/NS	102.4 (96.3, 108.9)	0.4435
	%FEV1 (%)	DT3.0a	298	95.9 (93.1, 99.0)						
		CC	97	91.0 (86.7, 95.6)	DT3.0a/CC	105.4 (99.5, 111.7)	0.0724			
		NS	92	96.2 (91.7, 101.0)	NS/CC	105.8 (98.7, 113.3)	0.1092	DT3.0a/NS	99.7 (94.2, 105.5)	0.9068
	FEV1% (%)	DT3.0a	298	79.4 (78.0, 80.8)						
		CC	97	78.0 (75.9, 80.2)	DT3.0a/CC	101.8 (98.5, 105.2)	0.3013			
		NS	92	81.0 (78.8, 83.3)	NS/CC	103.8 (99.8, 107.9)	0.0640	DT3.0a/NS	98.0 (94.9, 101.3)	0.2329
	FEF_25–75_ (L/s)	DT3.0a	298	2.8 (2.6, 3.0)						
		CC	97	2.5 (2.2, 2.8)	DT3.0a/CC	110.3 (96.8, 125.6)	0.1397			
		NS	92	2.8 (2.5, 3.2)	NS/CC	113.0 (96.7, 132.0)	0.1225	DT3.0a/NS	97.6 (85.8, 111.0)	0.71
	PEF (L/s)	DT3.0a	298	6.7 (6.4, 7.1)						
		CC	97	6.4 (5.9, 6.9)	DT3.0a/CC	105.2 (95.9, 115.5)	0.2804			
		NS	92	7.1 (6.5, 7.6)	NS/CC	110.4 (98.8, 123.3)	0.0809	DT3.0a/NS	95.4 (87.0, 104.5)	0.309

*2,3-d-TXB2* 2,3-dinor thromboxane B2, *8-epi-PGF2α* 8-iso prostaglandin F2α, *11-DHTXB2* 11-dehydrothromboxane B2, *CC* combustible cigarette, *CI* confidence interval, *DT3.0a* direct heating tobacco system platform 3 generation 3 version a, *FEF*_*25–75*_ forced expiratory flow between 25 and 75% of forced vital capacity, *FEV1* forced expiratory volume in one second, *FEV1%* percent forced expiratory volume in one second, *HDL-C* high-density lipoprotein cholesterol, *LS mean* least square mean, *NNAL* 4-(methylnitrosamino)−1-(3-pyridyl)−1-butanol, *NS* never smokers, *PEF* peak expiratory flow, *sICAM-1* soluble intercellular adhesion molecule-1, *WBC* white blood cell, *%FEV1* percent predicted forced expiratory volume in one second, *%FVC* percent predicted forced vital capacity

The NS group had significantly lower total NNAL levels than the CC group, with all NS group participants showing values below the limit of quantification. The DT3.0a group also had significantly lower total NNAL, at 60.9% (95% confidence interval [CI], 45.8–80.8%) of the CC group. As expected, total NNAL was significantly higher in the DT3.0a group compared to the NS group.

### Biomarkers of potential harm (BoPH)

The results for BoPH are presented in Table 4, with geometric LS mean ratios from the statistical model used to compare relative BoPH levels between the study groups.

Compared to the CC group, the NS group had significantly lower levels of 11-DHTXB2, 2,3-d-TXB2, and 8-epi-PGF2α at 78.2% (*p* = 0.0282), 67.8% (*p* = 0.0019), and 76.5% (*p* = 0.0155), respectively. Similarly, the DT3.0a group showed lower levels of these biomarkers relative to the CC group, with a significantly lower level of 2,3-d-TXB2 (80.6% of the CC group, *p* = 0.0402). In addition, the DT3.0a group showed higher levels of 11-DHTXB2, 2,3-d-TXB2, and 8-epi-PGF2α relative to the NS group, with a significant difference observed for 8-epi-PGF2α (121.2% of the NS group, *p* = 0.0362).

The NS group had significantly higher HDL-C than the CC group (124.4%, *p* = 0.0019). Similarly, the DT3.0a group showed significantly higher HDL-C than the CC group (132.0%, *p* < 0.0001). Compared with the NS group, the DT3.0a group also had higher HDL-C (106.1%) although the difference was not significant. The NS group had significantly lower WBC compared to the CC group (79.4%, *p* = 0.0012). Similarly, the DT3.0a group also showed lower WBC than the CC group, though the difference was not significant. The DT3.0a group had significantly higher WBC counts than the NS group (111.4%, *p* = 0.0284). For sICAM-1, the NS group showed a lower level (93.7%) than the CC group, but the difference was not significant. In contrast, the DT3.0a group showed significantly higher sICAM-1 levels compared with both the CC group (126.3%, *p* = 0.0011) and NS group (134.9%, *p* < 0.0001).

Compared with the CC group, the NS group showed higher values for all measured pulmonary functional parameters (%FVC, FEV1, %FEV1, FEV1%, FEF_25–75_, and PEF). Similarly, the DT3.0a group had higher values for all parameters relative to the CC group. When compared with the NS group, the DT3.0a group showed higher %FVC and FEV1, but lower %FEV1, FEV1%, FEF_25–75_, and PEF. There was no significant difference between the study groups for all pulmonary functional parameters.

### Relationship between BoPH and consumption

Linear regression analysis was performed to assess the relationship between BoPH and consumption, with the slopes and correlation coefficient of the regression lines presented in Table 5.

**Table 5:** Relationship between BoPH and product consumption

**Biomarker**	**Group**	***N***	**Slope (95% CI)**	**Correlation coefficient (Pearson r)**
HDL-C	DT3.0a	292	−0.0045 (−0.0084, −0.0005)	−0.1294*
	CC	95	−0.0053 (−0.0148, 0.0043)	−0.1131
sICAM-1	DT3.0a	292	0.0072 (0.0031, 0.0114)	0.1975*
	CC	95	0.0149 (0.0059, 0.0239)	0.3233*
WBC	DT3.0a	292	0.0032 (−0.0005, 0.0068)	0.0985
	CC	95	0.0134 (0.0046, 0.0222)	0.3003*
11-DHTXB2	DT3.0a	292	0.0012 (−0.0059, 0.0082)	0.0189
	CC	95	0.0138 (−0.0018, 0.0295)	0.1788
2,3-d-TXB2	DT3.0a	292	0.0124 (0.0048, 0.0200)	0.1842*
	CC	95	0.0209 (0.0052, 0.0366)	0.2649*
8-epi-PGF2α	DT3.0a	292	0.0033 (−0.0037, 0.0103)	0.0547
	CC	95	0.0189 (0.0069, 0.0308)	0.3091*
%FVC	DT3.0a	292	−0.0012 (−0.0030, 0.0007)	−0.0722
	CC	95	−0.0019 (−0.0061, 0.0022)	−0.0950
FEV1	DT3.0a	292	−0.0006 (−0.0036, 0.0024)	−0.0239
	CC	95	0.0001 (−0.0074, 0.0077)	0.0036
%FEV1	DT3.0a	292	−0.0010 (−0.0032, 0.0012)	−0.0531
	CC	95	−0.0018 (−0.0070, 0.0033)	−0.0742
FEV1%	DT3.0a	292	−0.0001 (−0.0014, 0.0013)	−0.0075
	CC	95	0.0004 (−0.0024, 0.0031)	0.0290
FEF_25–75_	DT3.0a	292	0.0001 (−0.0055, 0.0058)	0.0029
	CC	95	0.0020 (−0.0107, 0.0146)	0.0319
PEF	DT3.0a	292	−0.0013 (−0.0054, 0.0027)	−0.0376
	CC	95	−0.0057 (−0.0144, 0.0030)	−0.1333

^*^Statistically significant

*2,3-d-TXB2* 2,3-dinor thromboxane B2, *8-epi-PGF2α* 8-iso prostaglandin F2α, *11-DHTXB2* 11-dehydrothromboxane B2, *CC* combustible cigarette, *CI* confidence interval, *DT3.0a* direct heating tobacco system platform 3 generation 3 version a, *FEF*_*25–75*_ forced expiratory flow between 25 and 75% of forced vital capacity, *FEV1* forced expiratory volume in one second, *FEV1%* percent forced expiratory volume in one second, *HDL-C* high-density lipoprotein cholesterol, *NNAL* 4-(methylnitrosamino)−1-(3-pyridyl)−1-butanol, *PEF* peak expiratory flow, *sICAM-1* soluble intercellular adhesion molecule-1, *WBC* white blood cell, *%FEV1* percent predicted forced expiratory volume in one second, *%FVC* percent predicted forced vital capacity

In the CC group, significant slopes with consumption were observed for most blood and urine BoPH measures with the following slopes (95% CI): sICAM-1, 0.0149 (0.0059, 0.0239); WBC, 0.0134 (0.0046, 0.0222); 2,3-d-TXB2, 0.0209 (0.0052, 0.0366); and 8-epi-PGF2α, 0.0189 (0.0069, 0.0308). Slopes for HDL-C or 11-DHTXB2 were not significant with slopes (95% CI) of 0.0053 (− 0.0148, 0.0043) and 0.0138 (− 0.0018, 0.0295), respectively. The slopes for all pulmonary functional parameters were also not statistically significant.

In the DT3.0a group, significant slopes were observed for sICAM-1, 2,3-d-TXB2, and HDL-C, with slopes (95% CI) of 0.0072 (0.0031, 0.0114), 0.0124 (0.0048, 0.0200), and − 0.0045 (− 0.0084, − 0.0005), respectively. No significant slopes were found for WBC, 8-epi-PGF2α, and 11-DHTXB2 with slopes (95% CI) of 0.0032 (− 0.0005, 0.0068), 0.0033 (− 0.0037, 0.0103), and 0.0012 (− 0.0059, 0.0082), respectively. The slopes for all pulmonary functional parameters were also not statistically significant.

The absolute values of the slopes for all blood and urine BoPH were smaller in the DT3.0a group, while no consistent trend was observed when comparing slopes for pulmonary functional parameters between the two groups.

### Cough assessment

The results of the cough questionnaire are summarized in Table 6. For all scores, higher values indicate a favorable condition with respect to cough, with seven points as the maximum score for each of the physical, psychological, and social domains, and 21 points for the total LCQ score.

**Table 6:** Descriptive statistics of cough questionnaire

	Group	*N*	Mean (SD)	Median (min., max.)
Physical domain	DT3.0a	301	6.6 (0.5)	6.6 (3.9, 7.0)
	CC	98	6.4 (0.5)	6.6 (4.8, 7.0)
	NS	95	6.7 (0.3)	6.8 (5.4, 7.0)
Psychological domain	DT3.0a	301	6.8 (0.5)	7.0 (3.0, 7.0)
	CC	98	6.7 (0.5)	7.0 (5.1, 7.0)
	NS	95	6.9 (0.3)	7.0 (5.3, 7.0)
Social domain	DT3.0a	301	6.9 (0.5)	7.0 (1.5, 7.0)
	CC	98	6.8 (0.5)	7.0 (4.5, 7.0)
	NS	95	6.9 (0.4)	7.0 (4.3, 7.0)
LCQ	DT3.0a	301	20.2 (1.3)	20.6 (8.4, 21.0)
	CC	98	19.9 (1.3)	20.5 (14.8, 21.0)
	NS	95	20.4 (0.9)	20.8 (15.5, 21.0)

Scores were rated in a range from 1 to 7 for physical, psychological and social domains, and from 3 to 21 for LCQ score

*CC* combustible cigarette, *DT3.0a* direct heating tobacco system platform 3 generation 3 version a, *LCQ* the Leicester Cough Questionnaire, *NS* never smokers, *SD* standard deviation

Mean scores across all study groups ranged from 6.4 to 6.7 for the physical domain, 6.7–6.9 for the psychological domain, 6.8–6.9 for the social domain, and 19.9–20.4 for the total LCQ score. These scores were relatively high and closely aligned among the groups. For all domains, the NS group had higher mean scores than the CC group. For all domains, the DT3.0a group had higher mean scores than the CC group and similar or lower mean values than the NS group.

## Discussion

Previously published work showed that adults who smoked CC and switched to DT3.0a experienced substantial reductions in their exposure to the selected HPHCs found in CC smoke compared to those who continued to smoke CC, even after a short period of time [17]. The current study aimed to gain further insight into the potentially lower health risks associated with smoking in the DT3.0a use under real-world actual-use conditions, by assessing BoPH relevant to the health risks associated with smoking. It was designed as an observational, cross-sectional study in healthy adults who had used DT3.0a exclusively for more than 3 months. For comparison, the control groups comprised adults who had exclusively smoked CC for more than 1 year and those who had never smoked CCs.

The results of the exposure assessment showed that the total NNAL levels of all participants in the NS group were below the limit of quantification with the DT3.0a group exhibiting significantly lower total NNAL compared to the CC group (− 39.1%, *p* < 0.0037). This finding is consistent with a previous interventional study which reported reductions in total NNAL (− 36.3 or − 42.6%) over 5 days in adults who completely switched from CC to DT3.0a [17]. Given the similar magnitude of the reduction in total NNAL, a measure of tobacco-specific constituent exposure and an indicator of product use, these data support the likelihood that DT3.0a users in the present study also had lower exposure to the other selected HPHCs consistent with the reduction in NNK reported in the previous interventional study. This expectation is further supported by findings from the other interventional studies that measured exposure to a similar set of HPHCs, which showed that switching from CC to HTP reduced all BoEs, including total NNAL, within 5 days under confined settings, with the reductions maintained for an additional 85 days under ambulatory conditions [25, 26]. Consistent with the present results, other interventional studies in which adults switched from CC to HTP for several months also reported significant reductions in total NNAL compared with smokers who continued to use CCs [8, 24–26].

With regard to BoPH, in comparison with the CC group, the NS group showed higher HDL-C and pulmonary functional parameters, and lower levels of sICAM-1, WBC, 11-DHTXB2, 2,3-d-TXB2, and 8-epi-PGF2α. Significant differences were observed for all except sICAM-1 and pulmonary functional parameters. These observations are generally consistent with previous studies from other researchers [8–10].

In the DT3.0a group, HDL-C was significantly (*p* < 0.0001) higher than in the CC group but lower than in the NS group, though the difference with the NS group was not significant. The DT3.0a group also showed higher pulmonary functional parameters than the CC group, though the differences were not statistically significant. Compared with the NS group, pulmonary function parameters in the DT3.0a group was not markedly different with slightly higher %FVC and FEV1 and lower %FEV1, FEV1%, FEF_25–75_, and PEF. The DT3.0a group showed significantly lower 2,3-d-TXB2 (*p* = 0.0402) than the CC group, while the difference was not significant compared to the NS group. WBC, 11-DHTXB2, and 8-epi-PGF2α biomarkers in the DT3.0a group were also lower than in the CC group, but the difference was not significant. For sICAM-1, the DT3.0a group showed significantly higher levels than both the CC and NS groups (*p* = 0.0011 and *p* < 0.0001, respectively). Aside from the observation regarding sICAM-1, the overall data from the present study are consistent with the findings from other interventional studies comparing BoPH levels in adults who switched from CC to HTP, continued smoking CC, or abstained from smoking CC over several months [8, 24–26]. The directional consistency of favorable tendency of the BoPH as early indicators of diseases, reported in this study as consistent with the other clinical studies involving different HTPs [11, 26–28], indicates that by maintaining the principle of heating tobacco at 350 °C [29], which is below the reported 400 °C ignition temperature of tobacco [30, 31], and by ensuring HTPs are manufactured to high-quality and safety standards, products within the HTP category may have comparable potential to reduce the disease risk associated with smoking. In summary, the DT3.0a group generally exhibited BoPH profiles distinct from the CC group, with levels falling between those of the CC and NS groups, or, in the case of pulmonary functional parameters, not measurably different from the NS group. Taken together, these findings suggest that DT3.0a use is associated with favorable BoPH trends compared to CC smoking, potentially contributing to potentially lessened health impacts over time.

Previous interventional studies have reported that CC smoking groups have notably higher sICAM-1 than groups who quit smoking or never-smokers [32–34]. In contrast to these findings, no significant difference in sICAM-1 was observed between the CC and NS groups in the present study. In addition, sICAM-1 levels were higher in the DT3.0a group than in the CC group, which did not align with previous reports on HTP use [8, 24–26]. Moreover, the present sICAM-1 results showed different trends from the other inflammatory markers such as WBC and CRP (data not shown), as WBC and CRP levels in the DT3.0a group were lower than in the CC group but higher than in the NS group. These inconsistencies with previous findings, as well as with other relevant biomarkers within the same populations, suggest that some specific characteristics of sICAM-1 as a biomarker may have affected the present results. For example, previous studies have reported variability in sICAM-1 values. Lüdicke et al. assessed sICAM-1 in an interventional study in which adults who smoked were randomized to switch to HTP (mTHS group), continue smoking (mCC group) or abstain from smoking (SA group) for 90 days. In that study, the mTHS group had higher baseline sICAM-1 levels than the mCC group, but by Day 90, the values were nearly identical between the groups, leading to a significant reduction in sICAM-1 levels in the mTHS group. Moreover, in the same study, sICAM-1 levels were also higher in the SA group than in the mCC group at baseline and decreased to below the mCC group levels by Day 90. Another study reported short-term intra-individual variation in serum soluble adhesion molecules, including sICAM-1, ranging from 7.6 to 11.3%, highlighting the value of repeated measurements. Unlike these studies, the present cross-sectional design could not account for baseline levels or change in sICAM-1 levels over time. Therefore, given the reported variability of sICAM-1 over time, further evaluation and interpretation of sICAM-1 may be more appropriate in the context of change in values in longitudinal studies rather than those performed only by a single point assessment.

In adults who smoke, a dose–response relationship has been reported between BoPH levels and daily CC consumption, particularly for blood and urine BoPH [9]. In the present study, although daily consumption was similar between the CC group (13.9 ± 6.2 cigarettes/day) and the DT3.0a group (15.5 ± 7.7 sticks/day), favorable BoPH trends were observed in the DT3.0a group. Because DT3.0a generates an aerosol that is chemically less complex than CC smoke, with lower levels of HPHCs, the amount of daily DT3.0a use may be less relevant to BoPH levels. To investigate this, we performed an exploratory analysis of the dose–response relationship between BoPH levels and daily product consumption in the CC and DT3.0a groups. In the CC group, linear regression analysis showed positive slopes for sICAM-1, WBC, 2,3-d-TXB2, 8-epi-PGF2α, and 11-DHTXB2 and a negative slope for HDL-C, all consistent with previous findings [9]. In the DT3.0a group, the absolute values of these slopes were smaller than in the CC group, suggesting that daily product consumption has less impact on BoPH levels in DT3.0a use than in CC smoking. Combined with the favorable BoPH trends observed in the present study, these findings add to the evidence supporting that the health risks associated with smoking are potentially lower in DT3.0a use compared to CC smoking. For pulmonary functional parameters, no clear correlation with product consumption was observed in either the CC group or DT3.0a group. Previous studies have shown that FEV1 declines in adults who smoked CC, and that there were differences in FEV1 between adults who continued smoking and those who abstained [8–10]. It is noteworthy that such differences only emerged after more than 1 year. For FEF_25–75_, a negative correlation with pack-years has been reported [35]. These findings suggest that, for the pulmonary functional parameters, factors other than consumption, such as duration of tobacco product use, likely play an important role.

Analysis of the cough questionnaire showed the lowest mean scores in the CC group, with the NS group generally exhibiting higher scores. The DT3.0a group’s results were closer to those of the NS group. However, differences were not consistently evident across all measures. This can be attributed to the fact that all participants were healthy adults without noticeable symptoms.

### Limitations

The present study has several limitations, and results should be interpreted within this context. The cross-sectional, observational design compared separate populations that may have had different background factors such as alcohol consumption, physical activity, diet, or socioeconomic status. These factors could influence the measured BoPH but were not included as covariates in the statistical analysis. In addition, the outcomes provide only a snapshot at a single point in time. Longitudinal studies are needed to determine whether BoE and BoPH levels improve further over time (i.e., move closer to NS levels) in adults who switch from CC to DT3.0a. Tobacco product use status was classified based on self-reports, urinary cotinine, and exhaled CO, but potential misclassification cannot be excluded. Additional indicators should be considered to more accurately assess tobacco product use status over the medium to long term [36]. The present study focused on comparing the overall profile of various BoPH among the study groups, rather than prioritizing comparisons of individual BoPH between the groups. Due to this, adjustment for multiple comparisons was not considered for the statistical analysis, and the possibility of false-positive results cannot be ruled out. Notwithstanding these limitations, the present findings for exposure to NNK and various BoPH generally align with the results from other interventional studies conducted in different countries using other HTP.

## Conclusion

In the present study, exposure to NNK was lower in DT3.0a users who exclusively used the product under actual-use conditions compared to adults who smoke CCs. Most BoPH values in the DT3.0a group differed from those in the CC group, and showed trends similar to the NS group. The only exception was sICAM-1, which was inconsistent with previous reports and may warrant further reassessment in future longitudinal studies. Apart from this, DT3.0a use was associated with lower exposure to HPHCs and generally favorable BoPH profiles relative to CC smoking. In addition, daily DT3.0a consumption appeared to be less relevant to BoPH levels than CC consumption. Taking the present and previous studies into account, these findings add to the growing body of evidence that DT3.0a has the potential to reduce the health risks associated with smoking when used as an alternative to continued CC smoking by adults who smoke.

## Supplementary Information

Below is the link to the electronic supplementary material.

Supplementary file1 (DOCX 33 KB)

## Data Availability

As the data presented in this study contain information that could compromise the privacy of research participants, they are available from the corresponding author upon request.
