# Supplementary material for: Comparison of profiles of biomarkers of potential harm among healthy adults who used heated tobacco products, smoked combustible cigarettes, or who had never smoked: a cross-sectional, observational study
Source: Intern Emerg Med. 2025 Nov 26;21(2):497–510. doi: 10.1007/s11739-025-04202-z (PMC13061818; doi:10.1007/s11739-025-04202-z)
Supplement: Supplementary file 1 — Supplementary file1 (DOCX 33 KB) [file 11739_2025_4202_MOESM1_ESM.docx]

## **Supplementary figure 1 Disposition of participants**


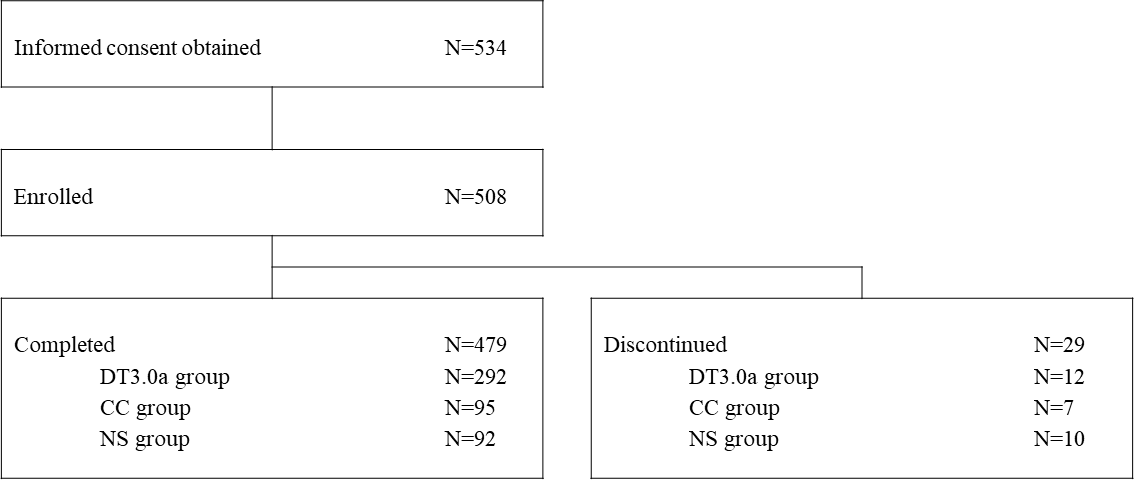


**Supplementary Figure 1 Disposition of participants**

CC, combustible cigarette; DT3.0a, direct heating tobacco system platform 3 generation 3 version a; NS, never smokers.

## Supplementary table 1 Descriptive statistics of biomarkers of exposure and potential harm

| **Biomarker [unit]** | **Group** | **N** | **Mean (SD)** | **Median (min., max.)** |
| --- | --- | --- | --- | --- |
| Total NNAL [ng/g creatinine] | DT3.0a | 292 | 43.5 (57.4) | 33.0 (1.3, 695.3) |
|  | CC | 95 | 94.5 (84.5) | 67.5 (3.2, 454.9) |
|  | NS | 92 | 2.3 (1.4) | 1.88 (0.7, 7.89) |
| HDL-C [mg/dL] | DT3.0a | 292 | 64.0 (17.3) | 62.0 (29.0, 125.0) |
|  | CC | 95 | 54.1 (17.3) | 51.0 (29.0, 112.0) |
|  | NS | 92 | 62.7 (18.6) | 59.5 (33.0, 115.0) |
| sICAM-1 [ng/mL] | DT3.0a | 292 | 517.8 (136.9) | 518.5 (192.0, 906.0) |
|  | CC | 95 | 449.1 (126.7) | 438.0 (207.0, 869.0) |
|  | NS | 92 | 418.9 (100.3) | 418.5 (244.0, 768.0) |
| WBC [10^3/uL] | DT3.0a | 292 | 6.2 (1.6) | 6.0 (3.2, 12.0) |
|  | CC | 95 | 6.9 (2.0) | 6.7 (3.1, 14.5) |
|  | NS | 92 | 5.7 (1.4) | 5.6 (2.0, 10.1) |
| 11-DHTXB2 [ng/g creatinine] | DT3.0a | 292 | 757.0 (467.2) | 679.2 (101.8, 5298.6) |
|  | CC | 95 | 964.2 (493.5) | 881.5 (255.5, 3759.6) |
|  | NS | 92 | 672.7 (255.4) | 648.0 (173.6, 1428.6) |
| 2,3-d-TXB2 [ng/g creatinine] | DT3.0a | 292 | 439.2 (308.7) | 371.5 (69.0, 4034.7) |
|  | CC | 95 | 585.0 (302.5) | 503.0 (153.9, 1664.9) |
|  | NS | 92 | 346.3 (156.2) | 307.3 (52.3, 715.7) |
| 8-epi-PGF2α [ng/g creatinine] | DT3.0a | 292 | 190.0 (109.4) | 167.5 (7.5, 1333.3) |
|  | CC | 95 | 205.8 (79.7) | 193.7 (64.1, 449.3) |
|  | NS | 92 | 153.4 (68.5) | 135.4 (44.9, 427.6) |
| %FVC [%] | DT3.0a | 298 | 101.0 (12.2) | 100.8 (57.9, 145.8) |
|  | CC | 97 | 97.1 (12.1) | 98.3 (66.8, 120.0) |
|  | NS | 92 | 100.2 (11.2) | 98.9 (74.5, 127.0) |
| FEV1 [L] | DT3.0a | 298 | 3.3 (0.6) | 3.3 (1.4, 5.0) |
|  | CC | 97 | 3.2 (0.7) | 3.2 (1.1, 5.0) |
|  | NS | 92 | 3.3 (0.6) | 3.3 (2.1, 4.6) |
| %FEV1 [%] | DT3.0a | 298 | 93.8 (12.8) | 93.5 (36.6, 135.8) |
|  | CC | 97 | 90.6 (12.8) | 90.8 (47.5, 117.1) |
|  | NS | 92 | 94.6 (11.6) | 93.5 (65.2, 127.0) |
| FEV1% [%] | DT3.0a | 298 | 79.4 (6.6) | 79.8 (52.7, 94.5) |
|  | CC | 97 | 79.9 (6.4) | 80.8 (59.2, 95.2) |
|  | NS | 92 | 80.9 (5.5) | 81.5 (64.9, 97.0) |
| FEF_25-75_ [L/s] | DT3.0a | 298 | 3.2 (1.1) | 3.1 (0.8, 6.9) |
|  | CC | 97 | 3.2 (1.0) | 3.2 (0.6, 6.7) |
|  | NS | 92 | 3.3 (0.9) | 3.3 (1.4, 5.5) |
| PEF [L/s] | DT3.0a | 298 | 8.0 (1.9) | 8.1 (1.9, 13.1) |
|  | CC | 97 | 7.7 (1.9) | 7.9 (2.9, 12.6) |
|  | NS | 92 | 8.2 (1.6) | 8.4 (3.6, 13.6) |

2,3-d-TXB2, 2,3-dinor thromboxane B2; 8-epi-PGF2α, 8-iso prostaglandin F2α; 11-DHTXB2, 11-dehydrothromboxane B2; CC, combustible cigarette; DT3.0a, direct heating tobacco system platform 3 generation 3 version a; FEF_25-75_, forced expiratory flow between 25% and 75% of forced vital capacity; FEV1, forced expiratory volume in one second; FEV1%, percent forced expiratory volume in one second; FVC, forced vital capacity; HDL-C, high-density lipoprotein cholesterol; LDL-C, low-density lipoprotein cholesterol; NNAL, 4-(methylnitrosamino)-1- (3-pyridyl)-1-butanol; NS, never smokers; PEF, peak expiratory flow; SD, standard deviation; sICAM-1, soluble intercellular adhesion molecule-1; TC, total cholesterol TG, triglyceride; WBC, white blood cell; %FEV1, percent predicted forced expiratory volume in one second; %FVC, percent predicted forced vital capacity.
